# Time Is Cerebellum

**DOI:** 10.1007/s12311-018-0925-6

**Published:** 2018-02-19

**Authors:** Hiroshi Mitoma, Mario Manto, Christiane S. Hampe

**Affiliations:** 1 Medical Education Promotion Center, Tokyo Medical University, Tokyo, Japan; 2 Unité d’Etude du Mouvement (UEM), FNRS, ULB-Erasme, 1070 Bruxelles, Belgium; 3 Service des Neurosciences, University of Mons, 7000 Mons, Belgium; 4 Department of Neurology, Centre Hospitalier Universitaire (CHU) de Charleroi, 6000 Charleroi, Belgium; 5 School of Medicine, University of Washington, Seattle, WA 98109 USA

## Abstract

The cerebellum characteristically has the capacity to compensate for and restore lost functions. These compensatory/restorative properties are explained by an abundant synaptic plasticity and the convergence of multimodal central and peripheral signals. In addition, extra-cerebellar structures contribute also to the recovery after a cerebellar injury. Clinically, some patients show remarkable improvement of severe ataxic symptoms associated with trauma, stroke, metabolism, or immune-mediated cerebellar ataxia (IMCA, e.g., multiple sclerosis, paraneoplastic cerebellar degeneration, gluten ataxia, anti-GAD65 antibody-associated cerebellar ataxia). However, extension of a cerebellar lesion can impact upon the fourth ventricle or the brainstem, either by direct or indirect mechanisms, leading to serious complications. Moreover, cerebellar reserve itself is affected by advanced cell loss and, at some point of disease progression, deficits become irreversible. Such phase transition from a treatable/restorable state (the reserve is still sufficient) to an untreatable state (the reserve is severely affected) is a loss of therapeutic opportunity, highlighting the need for early treatment during the restorable stage. Based on the motto of “Time is Brain,” a warning that stresses the importance of early therapeutic intervention in ischemic diseases, we propose “Time is Cerebellum” as a principle in the management of patients with cerebellar diseases, especially immune ataxias whose complexity often delay the therapeutic intervention. Indeed, this concept should not be restricted to ischemic cerebellar diseases. We argue that every effort should be made to reduce the diagnostic delay and to initiate early therapy to avoid the risk of transition from a treatable state to an irreversible condition and an associated accumulation of disability. The myriad of disorders affecting the cerebellum is a challenging factor that may contribute to irreversible disability if the window of therapeutic opportunity is missed.

“Time is Brain” was coined by Saver (2006) who proposed the importance of early intervention in ischemic brain diseases [1]. In stroke, the infarct core is surrounded by a functionally silent area (ischemic penumbra), which can be relieved by recanalization of blood flow [2]. Early intervention with thrombolytic therapy within 4.5 h has been recommended and is now applied worldwide [3]. Notably, Saver demonstrated a progressive damage of the brain tissue when such ischemic lesions are left temporally untreated. Based on meticulous evaluation, it was estimated that 120 million neurons, 83 billion synapses, and 714 km of myelinated fibers are lost each hour following occlusion of a typical large blood vessel [1]. These findings stress the notion that “pathologies become untreatable if treatment is not applied early during the disease onset, and more likely progress with associated clinical worsening.” In the discussion below, this feature is termed “phase transition” to highlight the shift from a treatable state to an irreversible condition.

We propose the concept “Time is Cerebellum” as a general principle in cerebellar diagnostic and therapeutic strategies. We emphasize that phase transition can be observed in many cerebellar pathologies encountered during daily practice. In this regard, the cerebellum is intrinsically capable of self-compensation and restoration. We have defined these capacities by coining the terminology of cerebellar reserve [4]. On the other hand, cerebellar reserve diminishes as the condition progresses during the natural history of the disorder. Consequently, a switch from an initial reversible stage to an irreversible stage occurs, with accumulation of disability. Early diagnosis and treatment are necessary in the management of patients with cerebellar ataxias (CAs). In other words, clinicians should not miss early treatment opportunities. This may appear obvious but it is not uncommon to see patients with CAs who are still left undiagnosed and untreated for weeks, months, or even years due to the complexity of cerebellar disorders and the myriad of entities. This editorial does not focus on the anatomical/functional changes which occur in extra-cerebellar structures implicated in the recovery after a cerebellar injury. Indeed, both compensatory and remote degenerative mechanisms may impact on the disease outcome [5].

## Cerebellar Reserve

**Fig. 1:**
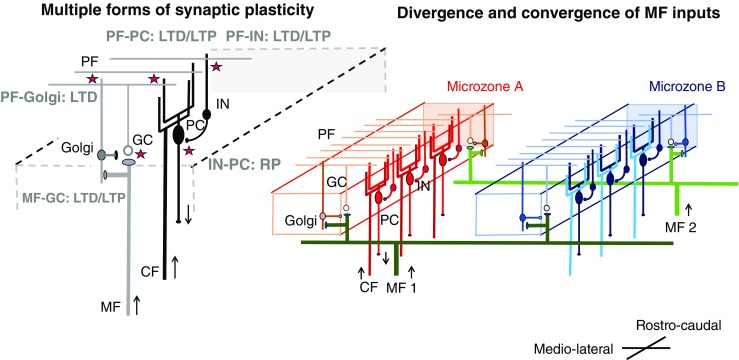
A schematic diagram of circuits in the cerebellar cortex. PC, Purkinje cell; GC, granule cell; IN, molecular layer interneurons; Golgi, Golgi cell; MF, mossy fiber; PF, parallel fiber; CF, climbing fiber. Two cellular mechanisms underlying cerebellar reserve are illustrated; (*1*) Multiple forms of synaptic plasticity and (*2*) convergence and divergence of mossy fiber inputs. *Multiple forms of synaptic plasticity*. Multiple forms of synaptic plasticity (illustrated by stars) co-exist in the cerebellar cortex [6, 7]. For example, conjunctive inputs from PF and CF on PC lead to depression of parallel fiber synapses [8]. LTD, long-term depression; LTP, long-term potentiation; RP, rebound potentiation. Excitatory neurons are shown with a white soma, whereas inhibitory neurons are shown with a black soma. *Convergence of inputs from different MF in one microzone and divergence of inputs from one MF to several microzones*. A group of CFs from a restricted area of the inferior olive nucleus innervates PCs which are located in a rostro-caudal sagittal area, forming a functional unit (microzone) [9]. MFs extend widely with a medio-lateral direction, terminating on GCs in different microzones [10]. For example, MF1 innervates both microzones A and B. Different MFs converge simultaneously to multiple microzones. For example, microzone A receives inputs from both MF1 and MF2. Thus, one microzone receives abundant central and peripheral inputs through MFs, which results in a redundancy of information. Most of them might be unutilized by a mechanism of silent synapse [11]. *Possible mechanisms underlying cerebellar reserve.* Damage to a single microzone can be compensated by other microzones reconstructing the lost internal model using synaptic plasticity and redundant central and peripheral information

**Fig. 2:**
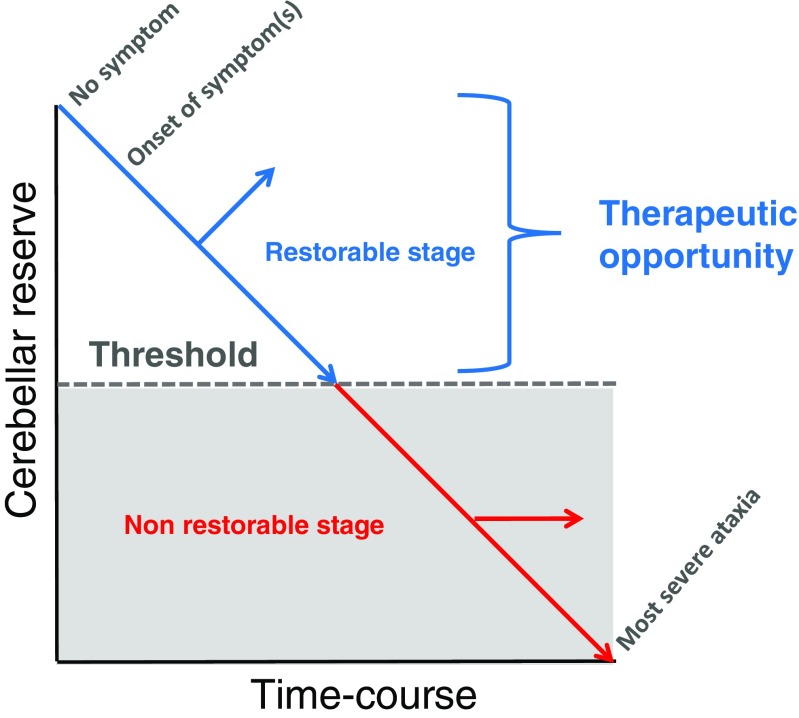
A schematic diagram of the decline of cerebellar function and the concept of “restorable stage” and “cerebellar reserve.” Proper therapies could restore cerebellar function in patients, whose cerebellum is at the “restorable stage,” meaning the presence of a sufficient reserve of cerebellar function. After a given threshold of neuronal loss or dysfunction in the cerebellar circuitry, cerebellar function cannot be restored anymore due to severe loss of computational capacity of the remaining cerebellar modules

Two categories of intrinsic mechanisms, characteristically embedded in the crystal-like cerebellar circuitry enriched in neurons, underlie cerebellar reserve: (*1*) several types of synaptic plasticity are active, and (*2*) there is a convergence of central and peripheral information into a given functional unit (microzone) within the cerebellum and into several microzones as a result of the modular organization of the cerebellum (Figs. 1 and 2).

In terms of plasticity, long-term synaptic depression (LTD) has been proposed as the synaptic mechanism of motor learning [8]. Conjunctive inputs from parallel fibers (PF) and climbing fibers (CF) on Purkinje cells lead to depression of parallel fiber synapses. It is hypothesized that CF-conveyed error signals eliminate PF-conveyed inadequate motor command signals [8]. Though still debated [6], synaptic plasticity appears to underlie the updating of the internal model after cerebellar damage. Through the updated internal model, the cerebellum can adapt to errors in executed movements and select the desired motor command in a predictive fashion [10].

Purkinje cells within a narrow rostro-caudal slit act as a functional unit. The mossy fibers (MFs; cerebellar inputs) extend widely in a medio-lateral fashion, terminating on granule cells [12]. PFs emerging from granule cells target the dendritic arborization of Purkinje neurons and the inhibitory neurons (the basket cells and the stellate cells). Based on this architecture, the MFs transmit simultaneously signals from the cerebral cortex or the periphery to multiple functional units. In other words, one cerebellar functional unit receives multiple central and peripheral inputs. This redundant information is used effectively to generate and adapt the internal model, thanks to synaptic plasticity.

## Phase Transition: from a Treatable to a Non-treatable State

In spite of the above-mentioned self-recovery capacities, some patients do not show recovery from CAs. In such cases, it is assumed that the pathology has advanced from a treatable state to an untreatable state. Such progression in treatability is determined by two factors.

First, the space in which the cerebellum is housed (the posterior fossa) becomes too small when the damaged cerebellum expands in volume in particular due to edematous changes. Provided the lesion (e.g., injury, hemorrhage, or cerebellitis) is limited to the cerebellum, the pathological process results in clinically evident CAs, without loss of consciousness or respiratory failure. The clinical condition of some of these patients has a high chance of improvement. On the other hand, edematous expansion of the lesion can cause a compression of adjacent structures, such as the fourth ventricle, due to the narrow anatomical space. This complication may induce an acute hydrocephalus, brainstem injury, and can even be fatal in some patients [4]. In summary, the anatomical features, the small space in the posterior fossa and closeness to the brainstem, explain the risk of development of expansion-related pathologies.

Second, there is a valid timeframe during which cerebellar reserve is preserved. Ample evidence suggests the existence of the restorable stage in immune-mediated cerebellar ataxias (IMCAs). IMCAs are a clinical entity established in the late 1980s and include multiple sclerosis (MS), paraneoplastic cerebellar degeneration, gluten ataxia, and anti-GAD65 antibody-associated cerebellar ataxia [13, 14]. Avoidance of antigens triggering the immune processes in some cases and administration of a combination of immunotherapies (e.g., corticosteroids, immunoglobulins, and immunosuppressants) are recommended [15]. Interestingly, although these immunotherapies can halt the progression of the immune-mediated attacks, the prognosis varies among the different types of IMACs. In some patients, especially those with no or only mild cerebellar atrophy, CAs can improve completely or partially from the clinical standpoint [15]. In contrast, other patients with evident cerebellar atrophy do not show any clinical improvement and exhibit a clinically stable CA [15]. This reflects the collapse of the dynamic capacity for compensation and restoration, mainly due to advanced cell death [16].

This scenario is mostly evident in anti-GAD65 antibody-associated CA [17]. Anti-GAD65 Ab acts on GAD65 and thus reduces GABA release by interfering with the packaging of this neurotransmitter into the synaptic terminal vesicles as well as shuttling of the vesicles to the synaptic cleft. The decrease in GABA subsequently attenuates spillover of GABA-induced presynaptic inhibition of glutamate release from neighboring synapses. Thus, anti-GAD65 Ab induces a major imbalance in neurotransmitters with an increase in glutamate levels and a decrease in GABA levels. The imbalance is also potentiated due to a positive feedback mechanism, since glutamate excess activates microglia through two mechanisms: non-synaptic release of glutamate through the xc(-)system and increase in neuroinflammatory factors (e.g., TNF-α), leading to inhibition of glutamate uptake via excitatory amino acid transporters (EAATs) on astrocytes [18]. Neuronal cell death is the ultimate consequence of continuous glutamate excess (excitotoxicity).

In the brain, there are 85–100 billions of neurons. About 60% of neurons are located in the cerebellum, even though the cerebellum constitutes only 10% of brain mass. The low ratio of glial cells to neurons in the cerebellum indicates a limited growth capacity for neurons and may explain their relative vulnerability. The loss of Purkinje cells will have a critical effect due to the relative low number of Purkinje cells (Purkinje cells ~ 15 × 10^6^) compared to granule cells (40 × 10^9^) [19, 20].

## To Avoid Missed Therapeutic Opportunities

In summary, although the cerebellum has high capacity for compensation and restoration, the clinical course of patients with cerebellar pathologies may include two types of phase transition to untreatable state: (*1*) extension of the lesion beyond the anatomic space of the cerebellum and (*2*) progression of pathology beyond the restoration stage. In order to avoid a missed opportunity (therapeutic window), careful monitoring of neurological manifestations and knowledge of natural history are of utmost importance in clinical ataxiology.

For the management of patients with potentially expanding cerebellar pathology (e.g., acute lesions accompanied by local tissue edema), it is important to protect the intact cerebellum and brainstem. Development of obstructive hydrocephalus requires immediate surgical treatment (ventricular drainage and/or suboccipital craniotomy) [4]. The caring physician should watch for clinical signs indicative of brainstem involvement, such as increased intracranial pressure, pyramidal signs, and impaired state of consciousness. These complications are very well established and daily management must take these risks into account.

When the clinician is facing patients showing CAs with chronic or insidious course, it is important to establish the correct diagnosis as soon as possible. Unfortunately, current clinical practice does not always follow this trivial recommendation. For instance, since the introduction of immunotherapies in the early stage of the disease (i.e., within the restorable stage at the time of preserved cerebellar reserve) is beneficial, differentiating IMCAs from alcohol-related cerebellar atrophy and degenerative CAs is of paramount importance. The presence of severe gait ataxia relative to the degree of cerebellar atrophy can provide a clue to the diagnosis of IMCAs [13, 14]. In the present circumstances, benefits in early intervention apply mainly to IMCAs. However, early interventions will be of growing importance in the near future, not only for IMCAs but also for all etiologies when molecular-targeting therapies [21] or neurotransplantation [22] becomes available in the clinic. In particular, animal experiments have shown that therapeutic modalities based on RNA interference (RNAi) and bioactive small molecules can suppress a particular gene and prevent cell death [21]. These techniques aim to prevent cell death in the early period. In addition, non-invasive cerebellar stimulation promotes synaptic plasticity and, therefore, this method has great potential for the reconstruction of cerebellar reserve [23]. Non-invasive stimulation may represent a promising strategy not only to improve the activity of residual cerebellar circuits but also as a complement tool to pharmacotherapy [23]. No doubt that the clinical benefits of such treatment modalities are expected to be more marked in patients with a sufficient cerebellar reserve.

In his classic paper of 1917, Holmes introduced the notion that the cerebellum has flexible repair capacities by describing a reversible clinical course in gunshot patients [24]. The monograph greatly influenced our understanding of the cerebellum. One hundred years later, the concept of “Time is Cerebellum” should push clinicians and neuroscientists to make every effort to reach a diagnosis and administer suitable therapies without delay, therefore preserving the cerebellar reserve to the maximum. Missing the therapeutic window can cause a disabling and irreversible condition (Table 1). A consensus effort should be made to define the ancillary examinations which should be performed within a very short timeframe to preserve neurons and improve functional outcomes. The search for validated surrogate markers of cerebellar ataxias remains a major objective.

**Table 1:** Time course and mechanisms of the most common cerebellar ataxias encountered in the clinic

Cerebellar disorder	Timing factor	Temporal window	Mechanisms
Trauma of the posterior fossa	Minutes/hours	*Trauma*, *hemorrhage*, *and cerebellitis*; during the period when the lesion is restricted to the cerebellum without compression of the brainstem. *Infarction*: during the period when the surrounding ischemic area is reversible	Bleeding Edema Inflammation Raised intracranial pressure
Cerebellar stroke	Minutes/hours		Ischemia Bleeding Raised intracranial pressure
Cerebellitis	Hours/days/weeks		Inflammation Raised intracranial pressure
Intoxication	Hours/days/weeks	During the period when there is no or only mild cerebellar atrophy (the degree of the cerebellar atrophy is morphologically correlated with that of loss in cerebellar reserve)	Abnormal synthesis of growth factors Impaired neurotransmission Excitotoxicity
Immune-mediated cerebellar ataxias (IMCAs)	Days/weeks/months		Auto-immune Inflammation Impaired neurotransmission
Alcohol-related cerebellar atrophy	Months/years		Excitotoxicity Endoplasmic reticulum stress Associated malnutrition Thiamine deficiency Deficits in growth factors
Multiple system atrophy	Months/years	In the present circumstances, there are still no effective therapies in preventing cell degeneration in primarily degenerative diseases of the cerebellum	Alpha-synucleinopathy
Hereditary degenerative cerebellar ataxias	Months/years		Neuronal inclusions Protein misfolding Impaired transcription
